# Psychedelic-like effects induced by 2,5-dimethoxy-4-iodoamphetamine, lysergic acid diethylamide, and psilocybin in male and female C57BL/6J mice

**DOI:** 10.1007/s00213-025-06795-x

**Published:** 2025-05-17

**Authors:** Shelby A. McGriff, Jacquelin C. Hecker, Alexander D. Maitland, John S. Partilla, Michael H. Baumann, Grant C. Glatfelter

**Affiliations:** 1 Designer Drug Research Unit, Intramural Research Program, National Institute on Drug Abuse, Baltimore, MD 21224 USA; 2 Behavioral Neuroscience Research Branch, National Institute on Drug Abuse Intramural Research Program, Baltimore, MD 21224 USA

**Keywords:** Psychedelics, Head twitch response, Mice, 5-HT_2A_, Sex

## Abstract

**Rationale:**

The head twitch response (HTR) is a spontaneously occurring behavior in mice that is increased in frequency by serotonergic psychedelics. The mouse HTR is often used as a proxy for psychedelic-like drug effects, but limited information is available about sex differences in HTRs evoked by various classes of psychedelics (i.e., phenethylamines, lysergamides, tryptamines).

**Objective and methods:**

To examine potential sex differences in responsiveness to structurally-distinct psychedelics, acute effects of subcutaneous 2,5-dimethoxy-4-iodo-amphetamine (DOI, 0.03–10 mg/kg), lysergic acid diethylamide (LSD, 0.003–1 mg/kg), and 4-phosphoryloxy-*N*,*N*-dimethyltryptamine (psilocybin, 0.03–10 mg/kg) on HTRs were compared in male and female C57BL/6J mice. For comparison, effects of the drugs on locomotor activity and body temperature were also determined.

**Results:**

Drug potencies for inducing HTRs were similar in males and females for all drugs, with only LSD exhibiting detectable differences due to increased maximal counts in females. Importantly, the maximum number of HTRs observed for all drugs was higher in females, with significant differences between sexes for DOI and LSD. Dose x sex interactions for the dose-response data were statistically significant for psilocybin and LSD, with females displaying more HTRs after the highest or peak doses of all drugs. The acute effects of drugs on locomotion and temperature varied by drug, but were similar in both sexes.

**Conclusions:**

The present results overall show no substantial sex differences in the potencies to induce HTRs for DOI, LSD, and psilocybin in C57BL/6J mice. However, females uniformly displayed more HTRs at high doses administered across chemotypes. The results further suggest that commonly used doses of psychedelics induce comparable psychedelic-like effects in male and female C57BL/6J mice, but modest differences may emerge at high doses.

**Supplementary Information:**

The online version contains supplementary material available at 10.1007/s00213-025-06795-x.

## Introduction

**Fig. 1:**
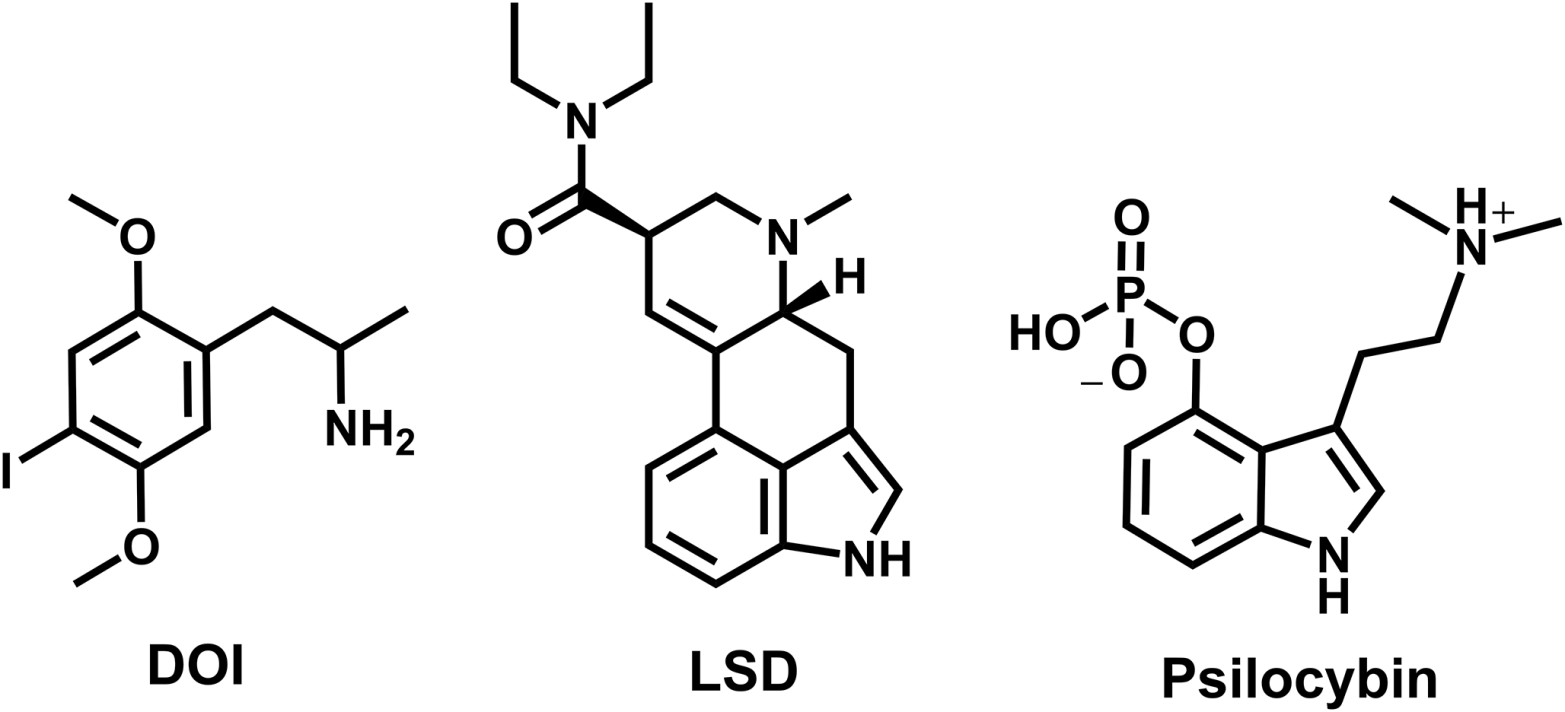
Chemical structures of standard phenethylamine (DOI), lysergamide (LSD), and tryptamine (psilocybin) serotonergic psychedelics tested in the study

Serotonergic psychedelic drugs produce subjective effects in humans, and psychedelic-like effects in rodents, via agonist actions at the serotonin 2 A receptor (5-HT_2A_)(Becker et al. 2023; Glatfelter et al. 2022b; González-Maeso et al. 2007; Halberstadt et al. 2011; Kometer et al. 2013; Vollenweider et al. 1998). Serotonergic psychedelics are often divided into three classes based on chemical structure: phenethylamines (e.g., 2,5-dimethoxy-4-iodoamphetamine or DOI), lysergamides (e.g., lysergic acid diethylamide or LSD), and tryptamines (e.g., 4-phosphoryloxy-*N*,* N*-dimethyltryptamine or psilocybin) (see chemical structures in Fig. 1)(Canal 2018; Kwan et al. 2022; Nichols 2016). While all three classes of serotonergic psychedelics are agonists at 5-HT_2A_, the other pharmacological targets for these chemotypes vary considerably. Phenethylamines generally display higher selectivity for 5-HT_2_ receptor subtypes vs. other receptors, whereas tryptamines and lysergamides are more “promiscuous”, interacting with a broad range of serotonergic (e.g., serotonin 1 A receptor or 5-HT_1A_) and non-serotonergic sites (Canal 2018; Glatfelter et al. 2023, 2024a; Kwan et al. 2022). From a clinical perspective, psychedelics are being investigated for their potential utility in treating a range of psychiatric disorders, such as depression, anxiety, substance use disorders (Johnson 2022; McClure-Begley and Roth 2022; Strickland and Johnson 2022), but the precise mechanisms underlying therapeutic efficacy are largely unknown. 

Phenethylamine, lysergamide, and tryptamine psychedelics reliably increase the head twitch response (HTR) in mice, a behavioral response which predicts potencies for psychedelic subjective effects of 5-HT_2A_ agonists in humans (Canal and Morgan 2012; Glatfelter et al. 2022b, 2024b; González-Maeso et al. 2007; Halberstadt et al. 2020; Klein et al. 2021; Sherwood et al. 2024). In particular, the potencies of drugs to increase HTRs in C57BL/6J mice are highly correlated with potencies to induce psychedelic subjective effects in humans (Halberstadt et al. 2020). Historically, most of the preclinical research examining behavioral effects of psychedelics has been carried out using only male subjects. In clinical studies, available evidence suggests no sex-related differences in various subjective ratings and other measures of acute drug effects of LSD and psilocybin (Aday et al. 2021; Ko et al. 2023; McCulloch et al. 2022; Vizeli et al. 2024), but data are limited by the narrow ranges of doses tested. Preclinically, studies reporting potential sex-related differences in acute, therapeutic, or other effects of psychedelics are emerging (Alper et al. 2023; Effinger et al. 2023, 2024; Herr and Baker 2020; McQueney and Garcia 2024; Meehan and Schechter 1998; Pálenícek et al. 2010; Rössler et al. 2006; Sierra et al. 2022; Tylš et al. 2016; Vohra et al. 2022). Regarding the HTR, one recent study reported dose-related sex differences for DOI-induced HTRs in C57BL/6J mice but not in 129S6/SvEv mice (Jaster et al. 2022), while an older study found no sex differences for DOI-induced HTRs in ICR mice (Darmani et al. 1996). A potential limitation of the aforementioned clinical and preclinical studies is the narrow ranges of doses tested. It is well established that the dose-response curves for psychedelic-induced HTRs exhibit a biphasic or inverted-U shape in mice (Brandt et al. 2016; Fantegrossi et al. 2010; Glatfelter et al. 2022a, b, 2023, 2024a; Halberstadt et al. 2020; Halberstadt and Geyer 2013; Klein et al. 2021; Sherwood et al. 2024), thereby highlighting the need for testing a range of doses to fully characterize potential sex differences in drug sensitivity. To date, such comparisons of drug potency and efficacy for induction of the HTR in male vs. female mice have not been reported and analogous clinical studies are likely not feasible.

Based on the limited number of studies evaluating sex differences in responsiveness to DOI and other psychedelics, we surmised that the effects of sex might be dose- and drug-dependent. Here, we first compared the binding affinities of DOI, LSD, and psilocin (the 4-hydroxy bioactive metabolite of psilocybin) at 5-HT_2A_ and 5-HT_1A_ in brain tissue from male and female mice. Next, the acute dose-related effects of DOI (0.03–10 mg/kg), LSD (0.003–1 mg/kg), and psilocybin (0.03–10 mg/kg) on HTRs were examined in male and female C57BL/6J mice. For comparison, we also examined the effects of the drugs on locomotor activity and body temperature. Using this approach, the potency and efficacy for three structurally-distinct serotonergic psychedelics (i.e., DOI, LSD, and psilocybin) were compared between age-matched male and female mouse cohorts. Importantly, this study is the first to evaluate potential sex differences in psychedelic-like effects across a wide range of doses for distinct psychedelic chemotypes.

## Materials and methods

### Drugs

(±)DOI hydrochloride was purchased from Cayman Chemical. Psilocybin (zwitterion), psilocin (freebase), and (+)-lysergic acid diethylamide (+)-tartrate (2:1) were generously provided by the National Institute on Drug Abuse (NIDA) Drug Supply Program (Rockville, MD, USA). For radioligand binding assays, DOI, psilocin, and LSD were prepared as 10 mM stock solutions in 100% DMSO. For the mouse experiments, DOI, psilocybin, and LSD were dissolved in 0.9% saline and administered subcutaneously (s.c.) in an injection volume of 0.01 mL/g body weight. Doses administered s.c. are expressed as the chemical form noted above.

### Radioligand binding in mouse brain membranes

Competition binding studies were conducted in mouse brain tissue as previously described (Glatfelter et al. 2022b). Briefly, the experiments utilized membranes prepared from whole C57BL/6 mouse brain minus cerebellum (BioIVT, Westbury, NY, USA). Stock solutions of DOI, psilocin, and LSD were diluted in assay buffer to yield 8 different concentrations per drug. We assessed the ability of test drugs to compete for [^3^H]M100907 binding at 5-HT_2A_ (1 nM) or [^3^H]8-OH-DPAT binding at 5-HT_1A_ (0.5 nM). Radioligands were purchased commercially (PerkinElmer, Boston, MA, USA). Nonspecific binding was determined using 10 µM ketanserin for 5-HT_2A_ and serotonin for 5-HT_1A_. All data represent 3 experiments performed in triplicate.

### Mouse behavioral studies

#### Animals

C57BL/6J mice were received from Jackson Laboratory (Bar Harbor, ME, USA) at 8 weeks of age and were acclimated for 1–2 weeks in the animal research facility of the NIDA Intramural Research Program (IRP) (Baltimore, MD, USA). Animal facilities were fully accredited by the Association for Assessment and Accreditation of Laboratory Animal Care, and all animal procedures were conducted in accordance with Animal Study Proposals approved by the Animal Care and Use Committee of the NIDA IRP. Mice were initially group housed 4 per cage during acclimation under a 12-h light-dark cycle (lights on at 0700 h). Food and water were available ad libitum.

#### Study design

Each drug was tested using groups of 12 mice per sex (i.e., 24 total mice per drug). Experimental sessions were conducted once every 1–2 weeks for 2 months per drug. Experimental sessions were spaced apart by at least 1 week to minimize the potential for tolerance to drug effects (Canal and Morgan 2012; Darmani et al. 1990a; de la Fuente Revenga et al. 2022). Experiments were conducted during the light phase (1300–1700 local time; lights on = 0700 in a 12/12 light/dark cycle).

All mice were injected s.c. with temperature transponders (14 × 2 mm, model IPTT-300, Avidity Science, Waterford Wisconsin, USA) under brief isoflurane immobilization, and were given at least 1 week to recover (Glatfelter et al. 2022b). Mice were single housed post-implant, and for the duration of the experiments, to protect their devices from being removed by cage mates. Temperature was measured noninvasively using a handheld reader which is sensitive to the transponder signals.

At 1–2 weeks prior to the beginning of experiments, mice were habituated to handling and receiving s.c. saline injections. On the day of an experiment, mice were acclimated to the testing room in their home cage for at least 30 min prior to the start of sessions. Prior to each session, mice were weighed, and resting body temperatures were recorded. Mice were then placed into experimental chambers for acclimation. Behavioral testing was carried out in TruScan mouse locomotor arenas equipped with photobeam arrays (Coulbourn Instruments, Holliston, MA, USA) that were modified with cylindrical inserts and transparent flooring to aid in semi-automated HTR detection as previously described (Glatfelter et al. 2022a). After 5 min of acclimation, basal body temperature was recorded followed by s.c. injection of drug or vehicle, and mice were returned to test chambers for 30 min. Dose ranges for each drug were 0.03–10 mg/kg for DOI, 0.03–10 mg/kg for psilocybin, and 0.003–1 mg/kg for LSD. Mice were randomly assigned to specific doses at the beginning of the experiment and received different doses at each session.

During each session, locomotor activity was quantified via photobeam tracking of distance traveled in cm. Overhead GoPro Hero Black 7 video recordings (120 fps, 960p resolution) were analyzed using a commercially available software package from Clever Sys Inc. (Reston, VA, USA) for the measurement of HTR activity. Mouse body temperature was also collected post-session to calculate temperature change values (basal - post session temperature reading).

### Statistical analysis

For the binding results, K_i_ affinity values for DOI, psilocin, and LSD were calculated based on previously determined K_d_ values for [^3^H]M100907 at 5-HT_2A_ (K_d_ = 0.35 nM) and [^3^H]8-OH-DPAT at 5-HT_1A_ (K_d_ = 1.03 nM). Concentration-response data were fit to a single site non-linear regression model. For the HTR studies, potencies were determined from the ascending limb of the dose-response curves using ED_50_ potency values from [agonist] vs. response (variable slope, four parameter) least squares fit non-linear regression analyses, whereas efficacy was defined by the top output parameter or E_max_ from the aforementioned analyses. Biphasic dose-response effects were depicted using bell-shaped nonlinear regression curves for LSD and psilocybin. Best-fit values for ED_50_ and E_max_ from non-linear regression analyses were compared by extra sum-of-squares *F* tests. Mean effects for each behavioral endpoint were compared using two-way ANOVA (dose x sex), followed by Sidak’s multiple comparisons post-hoc test. Mean effects for pooled mouse behavioral data were compared via Welch’s ANOVA with Dunnett’s T3 post-hoc test, comparing all doses to saline vehicle controls (0 mg/kg). Alpha was set to 0.05 for all analyses.

## Results

### Radioligand binding at 5-HT_2A_ and 5-HT_1A_ in mouse brain

Table S1 depicts the affinity values for DOI, LSD, and psilocin at 5-HT_2A_ labeled with [^3^H]M100907 and 5-HT_1A_ labeled with [^3^H]8-OH-DPAT in male and female mouse brain membranes. 5-HT_2A_ and 5-HT_1A_ binding affinities varied across the compounds (K_i_ range = 1.9–113 nM and 0.95-2,869 nM, respectively), but affinities for each drug were similar in males and females (Table S1, Figure S1). As expected, the 5-HT_2_ selective agonist DOI displayed much higher binding affinities at 5-HT_2A_ (females, K_i_ = 11 nM; males, K_i_ = 13 nM) when compared to its affinities at 5-HT_1A_ (females, K_i_ = 2,651 nM; males, K_i_ = 2,869 nM). Conversely, LSD exhibited high affinities for both 5-HT_2A_ (females, K_i_ = 1.9 nM; males, K_i_ = 2.3 nM) and 5-HT_1A_ (females, K_i_ = 0.95 nM; males, K_i_ = 1.02 nM). Psilocin exhibited mid-nM affinities for 5-HT_2A_ (females, Ki = 113 nM; males, Ki = 110 nM) and 5-HT_1A_ (females, K_i_ = 100 nM; males, K_i_ = 113 nM). Overall, these data demonstrate that the affinities of the test ligands for 5-HT_2A_ and 5-HT_1A_ are similar in male and female mouse brain.

### Dose-response and time-course for acute effects of DOI, LSD, and psilocybin in male and female mice

The primary goal of the study was to test for potential sex differences in psychedelic-like effects induced by DOI (0.03-10 mg/kg), LSD (0.003-1 mg/kg), and psilocybin (0.03-10 mg/kg) in C57BL/6J mice. To accomplish this, the compounds were administered s.c. to age-matched male and female mice at various doses to measure the acute effects on HTR, body temperature, and locomotor activity (Glatfelter et al. 2022a, b).

**Fig. 2:**
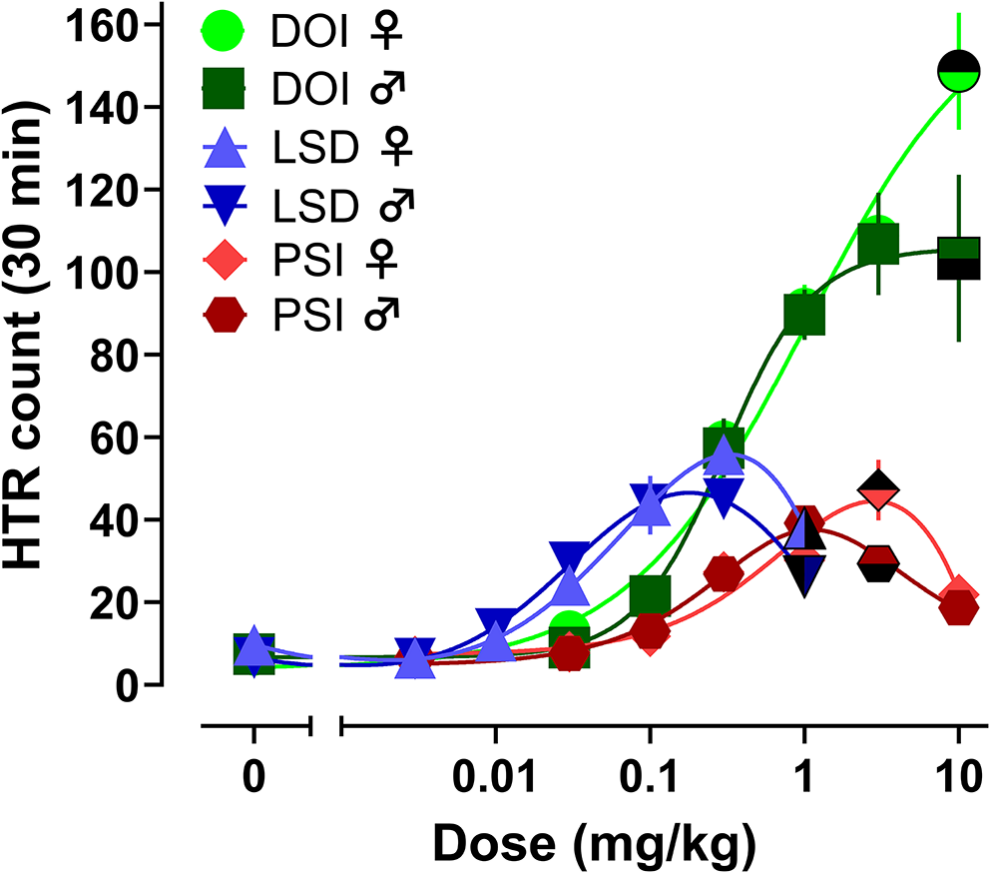
Dose-response curves for induction of the HTR by DOI (*n* = 5–7), LSD (*n* = 6), and psilocybin (*n* = 5–6) in male and female mice. Half-filled symbols represent significant dose-specific differences between sexes with accompanying statistical info in Table 1 and Table S2. Values represent mean HTR counts for each dose with SEM

**Table 1:** Potency (ED_50_) and maximum observed efficacy (E_max_) of DOI, LSD, and psilocybin for inducing the HTR in male and female mice. Potency and maximal values are expressed with 95% confidence intervals noted below in parentheses

Drug	Mouse Potency & Efficacy		
	HTR ♀	HTR ♂	HTR ♂+♀
	ED_50_ (95% CI) mg/kg s.c. E_max_ (95% CI) HTR events	ED_50_ (95% CI) mg/kg s.c. E_max_ (95% CI) HTR events	ED_50_ (95% CI) mg/kg s.c. E_max_ (95% CI) HTR events
DOI	0.52 (0.31–0.89) 131 (118–146)	0.30 (0.18–0.51) 105 (93–118)	0.33 (0.25–0.47) 111 (101–127)
LSD	0.051 (0.031–0.089) 58 (50–68)	0.024 (0.019–0.031) 46 (44–50)	0.035 (0.026–0.052) 51 (46–60)
Psilocybin	0.31 (0.14–0.90) 43 (35–54)	0.17 (0.09–0.31) 35 (30–40)	0.22 (0.14–0.40) 38 (33–47)

In male and female mice, all compounds produced dose-related changes in HTR, body temperature, and locomotor activity (see Fig. 2; Table 1, Table S2, Table S3, Table S4, Figure S2, Figure S3, Figure S4). The data depicted in Fig. 2 show that mean HTR counts increased in a dose-related manner compared to vehicle controls for each compound, with the dose-response curves for psilocybin and LSD following the typical inverted-U shape. Interestingly, for DOI doses > 1 mg/kg, HTR counts in males plateaued and began to descend while female counts did not. In the case of LSD, female HTR counts were nearly identical to male counts at low to moderate doses (0.003–0.1 mg/kg), but females displayed more HTRs at higher doses (0.3–1 mg/kg). For psilocybin, the female and male HTR counts were comparable at all doses, except for 3 mg/kg where females displayed more HTRs.

**Fig. 3:**
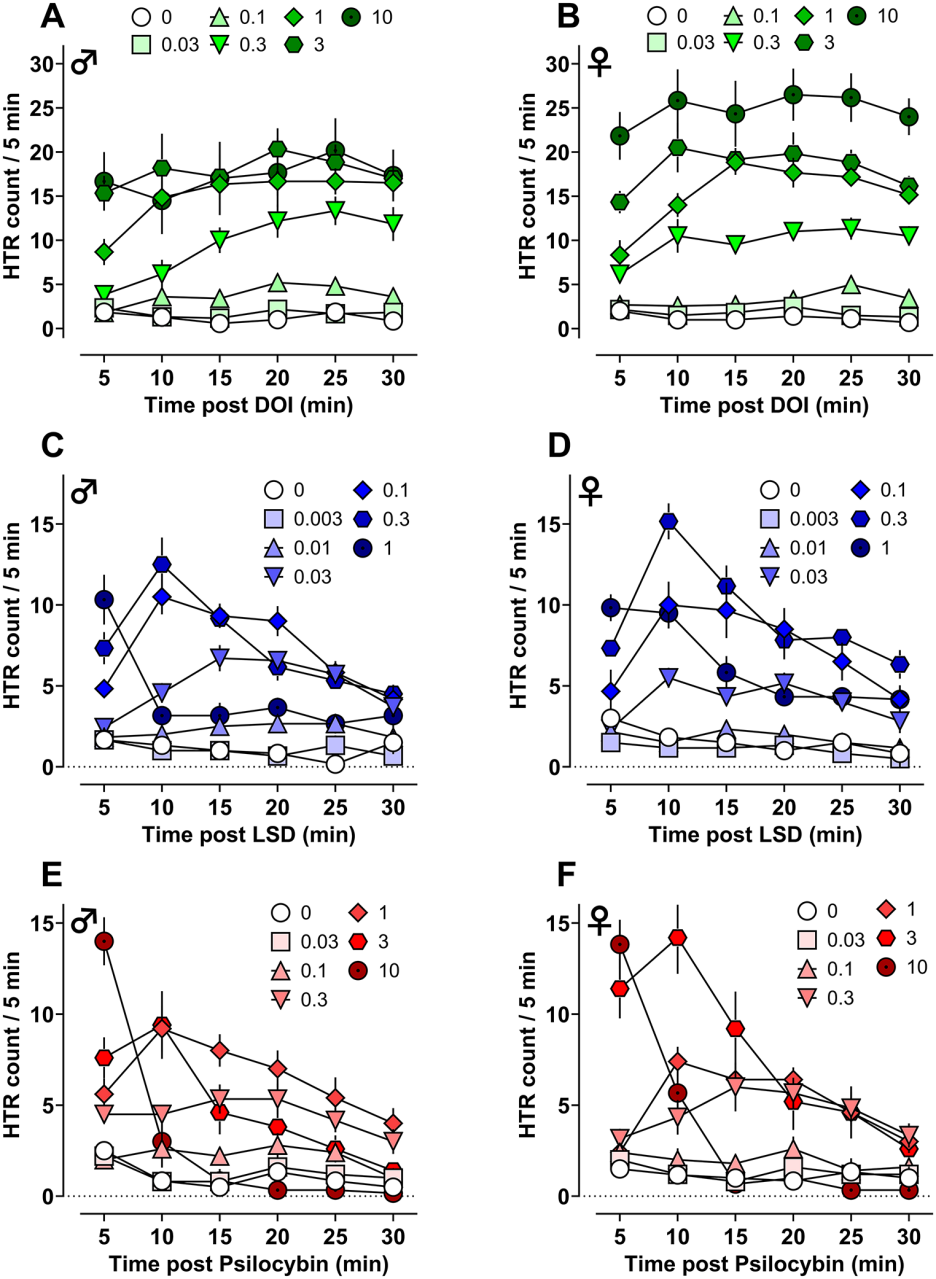
Time-course of dose-related effects for DOI (*n* = 5–7), LSD (*n* = 6 ), and psilocybin (*n* = 5–6) to induce HTR in male and female mice. Values represent mean HTR counts for each dose with SEM

For all drugs tested, the dose that produced peak HTR counts was right shifted in females, resulting in somewhat weaker drug potencies in females relative to males. For DOI, the ED_50_ = 0.52 mg/kg for females vs. 0.30 mg/kg for males; for LSD, ED_50_ = 0.051 mg/kg for females vs. 0.024 mg/kg for males; for psilocybin, ED_50_ = 0.31 mg/kg for females vs. 0.17 mg/kg for males (Fig. 2; Table 1). However, the confidence intervals for HTR potencies in females and males overlapped in all cases. HTR drug potencies were the same when compared using male and female regression models for DOI (*F*_1, 80_ = 2.456, *p* = 0.1288) and psilocybin (*F*_1, 58_ = 1.734, *p* = 0.1931), but potencies for LSD-induced HTR were different between regression models for each sex (*F*_1, 67_ = 7.545, *p* = 0.0077). Further, it is important to note that maximal HTR counts were higher in females relative to males across the three drugs. Comparison of best-fit top values (E_max_) from dose-response curves for regression models of male vs. female HTR counts revealed significant differences for DOI (E_max_ = 131 for females vs. 105 for males, *F*_1, 80_ = 8.450, *p* = 0.0047) and LSD (E_max_ = 58 for females vs. 46 for males, *F*_1, 67_ = 8.385, *p* = 0.0051), but not for psilocybin (E_max_ = 43 for females vs. 35 for males, *F*_1, 58_ = 3.191, *p* = 0.0793). The time-course for the effects of the compounds on HTR were similar between sexes, with DOI (Fig. 3A-B), LSD (Fig. 3C-D), and psilocybin (Fig. 3E-F) showing peak effects within 5–10 min post drug administration. Effects of DOI on HTR lasted throughout the entire 30 min session at peak doses, whereas for LSD and psilocybin, the increases in HTR at active doses trended to baseline levels by the end of the testing session.

Regarding acute temperature and locomotor effects of the drugs, DOI did not change body temperature vs. vehicle controls (Figure S3A & D) but did exhibit a marginal increase at the 1 mg/kg dose (Figure S4A & D). LSD produced mild hypothermia and decreased locomotion at the highest dose tested (1 mg/kg, Figure S3B & D, Figure S4B & D), but otherwise did not induce significant changes in these measures. At the two highest doses of psilocybin (3 & 10 mg/kg), significant hypothermia was observed (-1–4 °C, Figure S3C & D). Psilocybin administration produced mild increases in locomotor activity after doses of 0.3–1 mg/kg, but decreased locomotor activity at the highest dose of 10 mg/kg (Figure S4C & D). In general, the compounds displayed variable dose-related effects on body temperature motor and activity, but these acute effects were similar between males and females.

To statistically assess potential sex-related differences in effects of the drugs, the mean effect data depicted for each measure were compared using two-way ANOVA (dose x sex) followed by Sidak’s post hoc tests. These specific analyses were chosen based on the best practice statistical approaches outlined by Rich-Edwards and Maney (2023), which suggests using the interaction term for treatment x sex to test for male vs. female differences. There were significant dose x sex interactions for HTRs, with post-test differences; these sex differences in the dose-response for HTRs were driven by greater HTR counts in females after high-dose treatments (Fig. 2, Table S2, Table S3). Significant dose x sex interactions were observed for LSD (*F*_6, 71_ = 2.631, *p* = 0.0232) and psilocybin (*F*_6, 62_ = 2.683, *p* = 0.0222), but not for DOI (*F*_6, 72_ = 2.083, *p* = 0.0657) (Table S3). Post hoc analyses at each dose revealed sex differences in HTR counts between male and female subjects only at high doses of LSD (1 mg/kg, *p* = 0.0368), psilocybin (3 mg/kg, *p* = 0.0042), and DOI (10 mg/kg, *p* = 0.0017). Notably, these differences in responsiveness were observed at drug doses that were at least 10-fold greater than the ED_50_ doses for inducing HTR. No dose x sex interactions were observed for temperature or locomotor effects of the drugs (Table S4).

## Discussion

The main goal of the present study was to examine potential sex differences in responsiveness to DOI, LSD, and psilocybin in C57BL/6J mice. Our results highlight three major findings. First, binding affinities for DOI, LSD, and psilocin at mouse 5-HT_2A_ and 5-HT_1A_ were not different between males and females. Second, our in vivo studies demonstrated that all three psychedelic chemotypes produce dose-related increases in the HTR that are qualitatively similar in male and female mice, with comparable potencies across sexes. Third, we found that female mice exhibited greater maximal HTR counts than males after high-dose administration of all three psychedelics. Overall, the present data suggest that there are minimal sex differences in drug potencies for induction of HTR in C57BL/6J mice, but females may display more HTRs at high drug doses.

In our initial experiments, we evaluated binding affinities of the drugs at 5-HT_2A/1A_ receptor subtypes in whole brain membranes from C57BL/6J mice. In both males and females, DOI displayed low nanomolar affinity for [^3^H]M100907 binding at 5-HT_2A_ and µM affinity for [^3^H]8-OH-DPAT binding at 5-HT_1A_. By contrast, LSD and psilocin displayed nanomolar affinities (i.e., 1–100 nM) for both sites, in agreement with our previous findings in male mice (Glatfelter et al. 2022b, 2024c). The drug affinities that we observed in mouse brain membranes are consistent with K_i_ values reported by others who examined radioligand binding in cells transfected with human 5-HT_2A_ or 5-HT_1A_ (Janowsky et al. 2014; Nichols et al. 2002; Rickli et al. 2016). Our radioligand binding results provide no evidence for sex differences in drug affinities at 5-HT_2A_ or 5-HT_1A_ in mouse brain, but are limited without functional receptor assays and assessment of receptor levels between sexes. Addressing this and supporting our results, Jaster et al. previously showed that cortical 5-HT_2A_ signaling in response to DOI does not differ between sexes (Jaster et al. 2022). Nevertheless, prior research in rats shows that estrogen can increase 5-HT_2A_ receptor density (Sumner and Fink 1995, 1998) and expression (Birzniece et al. 2002; Cyr et al. 1998, 2000; Sumner et al. 2007) in various forebrain regions. In humans, estrogen administration may increase brain 5-HT_2A_ receptor binding in women (Kugaya et al. 2003; Moses et al. 2000). Therefore, there could be potential variability in 5-HT_2A_ expression levels in rodent and human brain related to sex. However, given the results from the present study finding no sex differences for measures of 5-HT_2A_ activities in mice, variability reported in previous studies for 5-HT_2A_ expression may not be pharmacologically relevant. It is also worth reiterating that all three serotonergic psychedelics tested have other receptor targets outside of 5-HT_2A_ and 5-HT_1A_ (e.g. 5-HT_2C_ receptors) which are implicated in their pharmacological effects (Fantegrossi et al. 2010; Glatfelter et al. 2022b; Nichols 2016). We included 5-HT_1A_ affinity comparisons due to the known influence of activity at this receptor to modulate 5-HT_2A_-mediated pharmacological effects clinically and preclinically (Brandt et al. 2018; Darmani et al. 1990b; Glatfelter et al. 2023, 2024a, b; Nichols 2016; Pokorny et al. 2016; Puigseslloses et al. 2024; Strassman 1995; Warren et al. 2024), but other receptors likely also play a role in overall pharmacological effects of the drugs.

In mouse behavioral experiments, we found that DOI, LSD, and psilocybin produced dose-related increases in HTRs in both males and females, with the same rank order of potencies observed across sexes (LSD > psilocybin ≥ DOI). By testing a wide range of doses, we found little evidence for sex differences in HTR drug potencies. Analyses of best-fit ED_50_ potency values from male vs. female regression models revealed that values were marginally different for LSD (i.e. likely did not come from the same model) but not for DOI or psilocybin. Despite differences observed for LSD-treated mice, mean HTR counts between sexes for ascending limb doses of all drugs overlapped, and no statistical differences in these values were detected. The ED_50_ values that were observed for DOI, LSD, and psilocybin after s.c. drug administration generally agree with those reported by us and other research groups using i.p. administration in male mice (Erkizia-Santamaría et al. 2022; Glatfelter et al. 2022a, b, 2024b; Halberstadt et al. 2020; Halberstadt and Geyer 2013). It is well established that the mouse HTR has predictive validity for drug effects in humans, with a strong positive correlation between drug potencies to induce HTRs in C57BL/6J mice and potencies to induce subjective psychedelic effects in humans (Halberstadt et al. 2020). Thus, our studies of full dose-response curves in mice align with and extend limited data showing no sex differences in the potencies for 5-HT_2A_ agonists to induce psychedelic subjective effects in humans.

The most important finding presented here is that minimal to no sex differences in HTR activity were observed at clinically relevant doses on the ascending limb of the HTR curve for each drug tested. However, the results indicate that researchers should carefully consider potential sex differences in HTR when using high drug doses. Female mice exhibited greater maximal HTRs than males after high-dose treatment with DOI, LSD, or psilocybin. Our statistical comparison of HTR counts between males and females revealed significant sex x dose interactions for LSD and psilocybin, with post-hoc tests only indicating sex differences specifically at high drug doses (i.e., drug doses at least 10-fold > than ED_50_ doses). The sex x dose interaction for DOI to induce HTRs did not reach statistical significance, but DOI also induced more HTRs in females at the highest dose administered. Furthermore, we found that E_max_ best-fit values for DOI and LSD were different between regression models for male vs. female mice. Our findings with DOI are consistent with those of Jaster et al. (2022), who reported a greater number of DOI-induced HTRs in female as compared to male C57BL/6J mice at a relatively high dose of 2 mg/kg. On the other hand, Darmani et al. (1996) detected no sex differences in responsiveness to DOI in albino ICR mice, though this strain appears to show fewer DOI-elicited HTRs relative to the C57BL/6J strain (Canal and Morgan 2012). Interestingly, Jaster et al. (2022) also observed that female mice had lower circulating levels of DOI when compared to male mice after i.p. drug administration, but found no sex-related differences in 5-HT_2A_-mediated second messenger activity in frontal cortex. Further, no sex differences were observed for HTR induced by DOI in 129S6/SvEv mice in the same study. Notably, only the C57BL/6J mouse strain has been validated for predicting relative potencies of serotonergic psychedelics in humans using the HTR (Halberstadt et al. 2020), and other strains have reduced responses to DOI (see Table 1 in Canal and Morgan 2012). At present, it is unclear if differences in relative maximal HTR counts are translatable to the magnitude of psychedelic subjective or other effects of 5-HT_2A_ agonists in humans, unlike relative drug potencies. Therefore, the differences observed at high doses of the drugs tested in mice may not reflect differences in effects of the drugs in humans. On the other hand though, comparing HTR maximal counts between structurally similar compounds can be useful to distinguish less psychoactive analogs with lower HTR counts from known psychoactive ones with higher relative HTR counts (Cunningham et al. 2023). Taken together, the findings across studies suggest the sex differences in sensitivity to DOI are strain- and dose-specific, whereby female C57BL/6J mice display more HTRs after high-dose drug administration. Our present findings extend prior work by showing greater HTR counts in female C57BL/6J mice after high-dose LSD and psilocybin, demonstrating that this phenomenon is generalizable across various psychedelic chemotypes.

Few previous studies have examined the impact of ovarian hormones on behavioral effects of psychedelics in rodents. Female rats in estrus and pro-estrus phases (i.e., when circulating levels of estradiol and progesterone are highest) are reported to display inhibited psilocin- and LSD-induced locomotor suppression, when compared to males and females in metestrus and diestrus phases (Pálenícek et al. 2010; Tylš et al. 2016). Miliano et al. (2019) examined the behavioral effects of a highly potent phenethylamine psychedelic in rats and showed the compound impairs pre-pulse inhibition (PPI) responding to the same extent in both sexes. By contrast, Vohra et al. (2022) demonstrated sex differences in PPI responses and startle amplitude induced by DOI and LSD in mice (Vohra et al. 2022). One notable limitation of the present study was the lack of estrus cycle monitoring, so it is not clear whether changes in ovarian hormones could modulate the behavioral effects of psychedelics on the serotonergic system. As such, future studies might examine the influence of estrus cyclicity, and the influence of estrogen administration, on the induction of the HTR and other effects of serotonergic psychedelics in female C57BL/6J mice.

A number of clinical studies have examined sex differences in the effects of psychedelics in human patients, and no substantial differences were found. For example, a recent metanalysis found no reported sex differences in the acute effects evoked by psychedelics (Aday et al. 2021). Clinical trial evidence confirms there are no sex differences in the intensity of mystical or challenging experiences with psilocybin, nor in the acute psychological or physiological effects of LSD (Ko et al. 2023; Vizeli et al. 2024). Nonetheless, clinical studies are limited in the number of doses and drugs that can be studied, as well as the ethical concerns regarding administration of high doses comparable to those associated with sex differences in mouse HTR. Thus, using the mouse HTR paradigm allowed us to address limitations of previous preclinical and clinical studies. The doses of psilocybin and LSD consumed by humans in recreational and clinical settings are much lower than the doses associated with sex differences shown here in mice, when the doses are compared directly without applying interspecies scaling. Psilocybin is consumed recreationally at doses ranging from 3 to > 35 mg (Erowid 2015). Early clinical trials using psilocybin for treatment of various neuropsychiatric indications administered oral doses ranging from 0.2 to 0.4 mg/kg (Garcia-Romeu et al. 2021), whereas more recent trials used a fixed oral dose of 25 mg (MacCallum et al. 2022). It is noteworthy that the clinically-administered doses of oral psilocybin noted above are close to the ED_50_ doses inducing HTR in C57BL/6J mice (i.e., 0.22–0.40 mg/kg, see Table 1, Glatfelter et al. 2022b, Sherwood et al. 2020). LSD is more potent than psilocybin, and it is consumed recreationally at lower doses ranging from 10 µg to > 400 µg (Erowid 2017). Clinical trials using LSD in healthy subjects generally administer a fixed dose of 200 µg (i.e., ~ 0.003 mg/kg for a 70 kg human) (Liechti 2017). Thus, unlike psilocybin, the clinically-administered doses of oral LSD are lower than the s.c. ED_50_ doses for inducing HTR in mice (i.e., 0.035–0.039 mg/kg, see Table 1 and Glatfelter et al. (2024b). The ED_50_ of LSD shown here and in our previous study with male mice translates to a ~ 2–3 mg dose in humans without interspecies scaling, which is an extremely high dose (Erowid 2017). This highlights the potential importance of other factors that may be driving species differences in drug potencies in some cases like LSD (e.g. pharmacokinetics, metabolism, mouse vs. human 5-HT_2A_ variant)(Dolder et al. 2015; Johnson et al. 1994; Kim et al. 2020). In summary, the typical oral doses of psilocybin and LSD administered to humans are far below the s.c. drug doses associated with the sex differences observed here in mice.

Given the increasing number of clinical trials testing the efficacy of psychedelics (Kurtz et al. 2022), along with the growing medication discovery efforts to identify new psychedelic-based treatments (McClure-Begley and Roth 2022), a more complete understanding of preclinical drug pharmacology and behavioral effects of the drugs between sexes is essential. High standards for study designs and statistical analyses to examine sex differences can be paramount for more rigorous and reproducible preclinical research (Becker et al. 2005; Rich-Edwards and Maney 2023). Utilizing these strategies, our present results expand the understanding of the nuanced preclinical behavioral effects of serotonergic psychedelic compounds in mice.

## Conclusion

The present data indicate negligible sex differences in the potencies for psychedelics to induce HTRs in C57BL/6J mice. However, female mice display greater maximal HTR counts compared to male mice at high doses of all three 5-HT_2A_ agonists tested. These data suggest the possibility that modest sex-related differences in the psychedelic-like effects of 5-HT_2A_ agonists may emerge after administration of high, non-clinically relevant drug doses.

## Electronic supplementary material

Below is the link to the electronic supplementary material.

Supplementary Material 1

## Data Availability

Summary data are provided in the supporting information tables and raw data are available from the corresponding author upon reasonable request.

## Abbreviations

5-HT: Serotonin
5-HT_1A_: Serotonin 1 A receptor
5-HT_2A_: Serotonin 2 A receptor
HTR: Head Twitch Response
DOI: 2,5-dimethoxy-4-iodoamphetamine
LSD: lysergic acid diethylamide
Psilocybin: 4-phosphoryloxy-*N*,*N*-dimethyltryptamine

## References

[CR1] Aday JS, Davis AK, Mitzkovitz CM, Bloesch EK, Davoli CC (2021) Predicting reactions to psychedelic drugs: A systematic review of States and traits related to acute drug effects. ACS Pharmacol Translational Sci 4:424–435 10.1021/acsptsci.1c00014 PMC8033773 33860172

[CR2] Alper K, Cange J, Sah R, Schreiber-Gregory D, Sershen H, Vinod KY (2023) Psilocybin sex-dependently reduces alcohol consumption in C57BL/6J mice. Front Pharmacol 13. 10.3389/fphar.2022.1074633 10.3389/fphar.2022.1074633 PMC9846572 36686713

[CR4] Becker JB, Arnold AP, Berkley KJ, Blaustein JD, Eckel LA, Hampson E, Herman JP, Marts S, Sadee W, Steiner M (2005) Strategies and methods for research on sex differences in brain and behavior. Endocrinology 146:1650–1673 15618360 10.1210/en.2004-1142

[CR3] Becker AM, Klaiber A, Holze F, Istampoulouoglou I, Duthaler U, Varghese N, Eckert A, Liechti ME (2023) Ketanserin reverses the acute response to LSD in a randomized, Double-Blind, Placebo-Controlled, crossover study in healthy participants. Int J Neuropsychopharmacol 26:97–106. 10.1093/ijnp/pyac075 36342343 10.1093/ijnp/pyac075 PMC9926053

[CR5] Birzniece V, Johansson I-M, Wang M-D, Bäckström T, Olsson T (2002) Ovarian hormone effects on 5-hydroxytryptamine2A and 5-hydroxytryptamine2C receptor mRNA expression in the ventral hippocampus and frontal cortex of female rats. Neurosci Lett 319:157–161 11834317 10.1016/s0304-3940(01)02570-8

[CR7] Brandt SD, Kavanagh PV, Westphal F, Stratford A, Elliott SP, Hoang K, Wallach J, Halberstadt AL (2016) Return of the lysergamides. Part I: analytical and behavioural characterization of 1-propionyl-d-lysergic acid diethylamide (1P-LSD). Drug Test Anal 8:891–902. 10.1002/dta.1884 26456305 10.1002/dta.1884 PMC4829483

[CR6] Brandt SD, Kavanagh PV, Twamley B, Westphal F, Elliott SP, Wallach J, Stratford A, Klein LM, McCorvy JD, Nichols DE, Halberstadt AL (2018) Return of the lysergamides. Part IV: analytical and Pharmacological characterization of lysergic acid morpholide (LSM-775). Drug Test Anal 10:310–322. 10.1002/dta.2222 28585392 10.1002/dta.2222 PMC6230476

[CR8] Canal CE (2018) Serotonergic psychedelics: experimental approaches for assessing mechanisms of action. Handb Exp Pharmacol 252:227–260. 10.1007/164_2018_107 29532180 10.1007/164_2018_107 PMC6136989

[CR9] Canal CE, Morgan D (2012) Head-twitch response in rodents induced by the hallucinogen 2, 5‐dimethoxy‐4‐iodoamphetamine: a comprehensive history, a re‐evaluation of mechanisms, and its utility as a model. Drug Test Anal 4:556–576 22517680 10.1002/dta.1333 PMC3722587

[CR10] Cunningham MJ, Bock HA, Serrano IC, Bechand B, Vidyadhara DJ, Bonniwell EM, Lankri D, Duggan P, Nazarova AL, Cao AB, Calkins MM, Khirsariya P, Hwu C, Katritch V, Chandra SS, McCorvy JD, Sames D (2023) Pharmacological mechanism of the Non-hallucinogenic 5-HT2A agonist Ariadne and analogs. ACS Chem Neurosci 14:119–135. 10.1021/acschemneuro.2c00597 36521179 10.1021/acschemneuro.2c00597 PMC10147382

[CR11] Cyr M, Bosse R, Di Paolo T (1998) Gonadal hormones modulate 5-hydroxytryptamine2A receptors: emphasis on the rat frontal cortex. Neuroscience 83:829–836 9483566 10.1016/s0306-4522(97)00445-4

[CR12] Cyr M, Landry M, Di Paolo T (2000) Modulation by estrogen-receptor directed drugs of 5-hydroxytryptamine-2A receptors in rat brain. Neuropsychopharmacology 23:69–78 10869887 10.1016/S0893-133X(00)00085-3

[CR13] Darmani NA, Martin BR, Glennon RA (1990a) Withdrawal from chronic treatment with (+/-)-DOI causes super-sensitivity to 5-HT2 receptor-induced head-twitch behaviour in mice. Eur J Pharmacol 186:115–118. 10.1016/0014-2999(90)94066-7 2282932 10.1016/0014-2999(90)94066-7

[CR14] Darmani NA, Martin BR, Pandey U, Glennon RA (1990b) Do functional relationships exist between 5-HT1A and 5-HT2 receptors? Pharmacol Biochem Behav 36:901–906. 10.1016/0091-3057(90)90098-3 2145593 10.1016/0091-3057(90)90098-3

[CR15] Darmani NA, Shaddy J, Gerdes CF (1996) Differential ontogenesis of three DOI-Induced behaviors in mice. Physiol Behav 60:1495–1500. 10.1016/S0031-9384(96)00323-X 8946497 10.1016/s0031-9384(96)00323-x

[CR16] de la Fuente Revenga M, Jaster AM, McGinn J, Silva G, Saha S, González-Maeso J (2022) Tolerance and Cross-Tolerance among psychedelic and nonpsychedelic 5-HT(2A) receptor agonists in mice. ACS Chem Neurosci 13:2436–2448. 10.1021/acschemneuro.2c00170 35900876 10.1021/acschemneuro.2c00170 PMC10411500

[CR17] Dolder PC, Schmid Y, Haschke M, Rentsch KM, Liechti ME (2015) Pharmacokinetics and Concentration-Effect relationship of oral LSD in humans. Int J Neuropsychopharmacol 19. 10.1093/ijnp/pyv072 10.1093/ijnp/pyv072 PMC4772267 26108222

[CR19] Effinger DP, Quadir SG, Ramage MC, Cone MG, Herman MA (2023) Sex-specific effects of psychedelic drug exposure on central amygdala reactivity and behavioral responding. Transl Psychiatry 13:119. 10.1038/s41398-023-02414-5 37031219 10.1038/s41398-023-02414-5 PMC10082812

[CR18] Effinger DP, Hoffman JL, Mott SE, Magee SN, Quadir SG, Rollison CS, Toedt D, Echeveste Sanchez M, High MW, Hodge CW, Herman MA (2024) Increased reactivity of the paraventricular nucleus of the hypothalamus and decreased threat responding in male rats following Psilocin administration. Nat Commun 15:5321. 10.1038/s41467-024-49741-9 38909051 10.1038/s41467-024-49741-9 PMC11193716

[CR20] Erkizia-Santamaría I, Alles-Pascual R, Horrillo I, Meana JJ, Ortega JE (2022) Serotonin 5-HT2A, 5-HT2c and 5-HT1A receptor involvement in the acute effects of psilocybin in mice. In vitro Pharmacological profile and modulation of thermoregulation and head-twich response. Biomed Pharmacother 154:113612. 10.1016/j.biopha.2022.113612 36049313 10.1016/j.biopha.2022.113612

[CR21] Erowid (2015) Psilocybin vault. Psilocybin & Psilocin Dosage

[CR22] Erowid (2017) LSD (Acid) vault. LSD Dosage

[CR23] Fantegrossi WE, Simoneau J, Cohen MS, Zimmerman SM, Henson CM, Rice KC, Woods JH (2010) Interaction of 5-HT2A and 5-HT2C receptors in R(-)-2,5-dimethoxy-4-iodoamphetamine-elicited head twitch behavior in mice. J Pharmacol Exp Ther 335:728–734. 10.1124/jpet.110.172247 20858706 10.1124/jpet.110.172247 PMC2993545

[CR24] Garcia-Romeu A, Barrett FS, Carbonaro TM, Johnson MW, Griffiths RR (2021) Optimal dosing for psilocybin pharmacotherapy: considering weight-adjusted and fixed dosing approaches. J Psychopharmacol 35:353–361. 10.1177/0269881121991822 33611977 10.1177/0269881121991822 PMC8056712

[CR25] Glatfelter GC, Chojnacki MR, McGriff SA, Wang T, Baumann MH (2022a) Automated computer software assessment of 5-Hydroxytryptamine 2A Receptor-Mediated head twitch responses from video recordings of mice. ACS Pharmacol Translational Sci. 10.1021/acsptsci.1c00237 10.1021/acsptsci.1c00237 PMC9112414 35592434

[CR28] Glatfelter GC, Pottie E, Partilla JS, Sherwood AM, Kaylo K, Pham DNK, Naeem M, Sammeta VR, DeBoer S, Golen JA, Hulley EB, Stove CP, Chadeayne AR, Manke DR, Baumann MH (2022b) Structure-Activity relationships for psilocybin, Baeocystin, aeruginascin, and related analogues to produce Pharmacological effects in mice. ACS Pharmacol Transl Sci 5:1181–1196. 10.1021/acsptsci.2c00177 36407948 10.1021/acsptsci.2c00177 PMC9667540

[CR27] Glatfelter GC, Naeem M, Pham DNK, Golen JA, Chadeayne AR, Manke DR, Baumann MH (2023) Receptor binding profiles for tryptamine psychedelics and effects of 4-Propionoxy-N,N-dimethyltryptamine in mice. ACS Pharmacol Translational Sci. 10.1021/acsptsci.2c00222 10.1021/acsptsci.2c00222 PMC10111620 37082754

[CR26] Glatfelter GC, Clark AA, Cavalco NG, Landavazo A, Partilla JS, Naeem M, Golen JA, Chadeayne AR, Manke DR, Blough BE, McCorvy JD, Baumann MH (2024a) Serotonin 1A receptors modulate serotonin 2A Receptor-Mediated behavioral effects of 5-Methoxy-N,N-dimethyltryptamine analogs in mice. ACS Chem Neurosci. 10.1021/acschemneuro.4c00513 39636099 10.1021/acschemneuro.4c00513 PMC12745965

[CR29] Glatfelter GC, Pottie E, Partilla JS, Stove CP, Baumann MH (2024b) Comparative Pharmacological effects of lisuride and lysergic acid diethylamide revisited. ACS Pharmacol Translational Sci 7:641–653. 10.1021/acsptsci.3c00192 10.1021/acsptsci.3c00192 PMC10928901 38481684

[CR30] Glatfelter GC, Pottie E, Partilla JS, Stove CP, Baumann MH (2024c) Comparative Pharmacological effects of lisuride and lysergic acid diethylamide revisited. ACS Pharmacology & Translational Science 10.1021/acsptsci.3c00192 PMC10928901 38481684

[CR31] González-Maeso J, Weisstaub NV, Zhou M, Chan P, Ivic L, Ang R, Lira A, Bradley-Moore M, Ge Y, Zhou Q, Sealfon SC, Gingrich JA (2007) Hallucinogens recruit specific cortical 5-HT2A Receptor-Mediated signaling pathways to affect behavior. Neuron 53:439–452. 10.1016/j.neuron.2007.01.008 17270739 10.1016/j.neuron.2007.01.008

[CR33] Halberstadt AL, Geyer MA (2013) Characterization of the head-twitch response induced by hallucinogens in mice: detection of the behavior based on the dynamics of head movement. Psychopharmacology 227:727–739. 10.1007/s00213-013-3006-z 23407781 10.1007/s00213-013-3006-z PMC3866102

[CR34] Halberstadt AL, Koedood L, Powell SB, Geyer MA (2011) Differential contributions of serotonin receptors to the behavioral effects of indoleamine hallucinogens in mice. J Psychopharmacol 25:1548–1561. 10.1177/0269881110388326 21148021 10.1177/0269881110388326 PMC3531560

[CR32] Halberstadt AL, Chatha M, Klein AK, Wallach J, Brandt SD (2020) Correlation between the potency of hallucinogens in the mouse head-twitch response assay and their behavioral and subjective effects in other species. Neuropharmacology 167:107933 31917152 10.1016/j.neuropharm.2019.107933 PMC9191653

[CR35] Herr KA, Baker LE (2020) Re-evaluation of the discriminative stimulus effects of lysergic acid diethylamide with male and female Sprague-Dawley rats. Behav Pharmacol 31:776–786. 10.1097/fbp.0000000000000589 32960851 10.1097/FBP.0000000000000589

[CR36] Janowsky A, Eshleman AJ, Johnson RA, Wolfrum KM, Hinrichs DJ, Yang J, Zabriskie TM, Smilkstein MJ, Riscoe MK (2014) Mefloquine and psychotomimetics share neurotransmitter receptor and transporter interactions in vitro. Psychopharmacology 231:2771–2783. 10.1007/s00213-014-3446-0 24488404 10.1007/s00213-014-3446-0 PMC4097020

[CR37] Jaster AM, Younkin J, Cuddy T, de la Fuente Revenga M, Poklis JL, Dozmorov MG, González-Maeso J (2022) Differences across sexes on head-twitch behavior and 5-HT(2A) receptor signaling in C57BL/6J mice. Neurosci Lett 788:136836. 10.1016/j.neulet.2022.136836 35963476 10.1016/j.neulet.2022.136836 PMC10114867

[CR39] Johnson MW (2022) Classic psychedelics in addiction treatment: the case for psilocybin in tobacco smoking cessation. Curr Top Behav Neurosci. 10.1007/7854_2022_327 35704271 10.1007/7854_2022_327

[CR38] Johnson MP, Loncharich RJ, Baez M, Nelson DL (1994) Species variations in transmembrane region V of the 5-hydroxytryptamine type 2A receptor alter the structure-activity relationship of certain Ergolines and tryptamines. Mol Pharmacol 45:277–286 8114677

[CR40] Kim K, Che T, Panova O, DiBerto JF, Lyu J, Krumm BE, Wacker D, Robertson MJ, Seven AB, Nichols DE, Shoichet BK, Skiniotis G, Roth BL (2020) Structure of a Hallucinogen-Activated Gq-Coupled 5-HT(2A) serotonin receptor. Cell 182:1574–1588e19. 10.1016/j.cell.2020.08.024 32946782 10.1016/j.cell.2020.08.024 PMC7593816

[CR41] Klein AK, Chatha M, Laskowski LJ, Anderson EI, Brandt SD, Chapman SJ, McCorvy JD, Halberstadt AL (2021) Investigation of the Structure-Activity relationships of psilocybin analogues. ACS Pharmacol Transl Sci 4:533–542. 10.1021/acsptsci.0c00176 33860183 10.1021/acsptsci.0c00176 PMC8033608

[CR42] Ko K, Carter B, Cleare AJ, Rucker JJ (2023) Predicting the Intensity of Psychedelic-Induced Mystical and Challenging Experience in a Healthy Population: An Exploratory Post-Hoc Analysis. Neuropsychiatric Disease and Treatment: 2105–2113 10.2147/NDT.S426193 PMC10561760 37818448

[CR43] Kometer M, Schmidt A, Jäncke L, Vollenweider FX (2013) Activation of serotonin 2A receptors underlies the psilocybin-induced effects on α oscillations, N170 visual-evoked potentials, and visual hallucinations. J Neurosci 33:10544–10551. 10.1523/jneurosci.3007-12.2013 23785166 10.1523/JNEUROSCI.3007-12.2013 PMC6618596

[CR44] Kugaya A, Epperson CN, Zoghbi S, Van Dyck CH, Hou Y, Fujita M, Staley JK, Garg PK, Seibyl JP, Innis RB (2003) Increase in prefrontal cortex serotonin2A receptors following Estrogen treatment in postmenopausal women. Am J Psychiatry 160:1522–1524 12900319 10.1176/appi.ajp.160.8.1522

[CR45] Kurtz JS, Patel NA, Gendreau JL, Yang C, Brown N, Bui N, Picton B, Harris M, Hatter M, Beyer R (2022) The use of psychedelics in the treatment of medical conditions: an analysis of currently registered psychedelics studies in the American drug trial registry. Cureus 14 10.7759/cureus.29167 PMC9567237 36259015

[CR46] Kwan AC, Olson DE, Preller KH, Roth BL (2022) The neural basis of psychedelic action. Nat Neurosci 25:1407–1419. 10.1038/s41593-022-01177-4 36280799 10.1038/s41593-022-01177-4 PMC9641582

[CR47] Liechti ME (2017) Modern clinical research on LSD. Neuropsychopharmacology 42:2114–2127 28447622 10.1038/npp.2017.86 PMC5603820

[CR48] MacCallum CA, Lo LA, Pistawka CA, Deol JK (2022) Therapeutic use of psilocybin: practical considerations for dosing and administration. Front Psychiatry 13:1040217 36532184 10.3389/fpsyt.2022.1040217 PMC9751063

[CR49] McClure-Begley TD, Roth BL (2022) The promises and perils of psychedelic Pharmacology for psychiatry. Nat Rev Drug Discov. 10.1038/s41573-022-00421-7 35301459 10.1038/s41573-022-00421-7

[CR50] McCulloch DE-W, Grzywacz MZ, Madsen MK, Jensen PS, Ozenne B, Armand S, Knudsen GM, Fisher PM, Stenbæk DS (2022) Psilocybin-induced mystical-type experiences are related to persisting positive effects: A quantitative and qualitative report. Front Pharmacol 13:841648 35355714 10.3389/fphar.2022.841648 PMC8959755

[CR51] McQueney AJ, Garcia EJ (2024) Biological sex modulates the efficacy of 2,5-dimethoxy-4-iodoamphetamine (DOI) to mitigate Fentanyl demand. Drug Alcohol Depend 263:112426. 10.1016/j.drugalcdep.2024.112426 39217832 10.1016/j.drugalcdep.2024.112426 PMC12952523

[CR52] Meehan SM, Schechter MD (1998) LSD produces conditioned place preference in male but not female Fawn hooded rats. Pharmacol Biochem Behav 59:105–108. 10.1016/S0091-3057(97)00391-2 9443543 10.1016/s0091-3057(97)00391-2

[CR53] Miliano C, Marti M, Pintori N, Castelli MP, Tirri M, Arfè R, De Luca MA (2019) Neurochemical and behavioral profiling in male and female rats of the psychedelic agent 25I-NBOMe. Front Pharmacol 10:1406 31915427 10.3389/fphar.2019.01406 PMC6921684

[CR54] Moses EL, Drevets WC, Smith G, Mathis CA, Kalro BN, Butters MA, Leondires MP, Greer PJ, Lopresti B, Loucks TL (2000) Effects of estradiol and progesterone administration on human serotonin 2A receptor binding: a PET study. Biol Psychiatry 48:854–860 11063980 10.1016/s0006-3223(00)00967-7

[CR55] Nichols DE (2016) Psychedelics Pharmacol Reviews 68:264–355. 10.1124/pr.115.011478 10.1124/pr.115.011478 PMC4813425 26841800

[CR56] Nichols DE, Frescas S, Marona-Lewicka D, Kurrasch-Orbaugh DM (2002) Lysergamides of isomeric 2,4-dimethylazetidines map the binding orientation of the diethylamide moiety in the potent hallucinogenic agent N,N-diethyllysergamide (LSD). J Med Chem 45:4344–4349. 10.1021/jm020153s 12213075 10.1021/jm020153s

[CR57] Pálenícek T, Hlinák Z, Bubeníková-Valesová V, Novák T, Horácek J (2010) Sex differences in the effects of N,N-diethyllysergamide (LSD) on behavioural activity and prepulse Inhibition. Prog Neuropsychopharmacol Biol Psychiatry 34:588–596. 10.1016/j.pnpbp.2010.02.008 20156516 10.1016/j.pnpbp.2010.02.008

[CR58] Pokorny T, Preller KH, Kraehenmann R, Vollenweider FX (2016) Modulatory effect of the 5-HT1A agonist Buspirone and the mixed non-hallucinogenic 5-HT1A/2A agonist ergotamine on psilocybin-induced psychedelic experience. Eur Neuropsychopharmacol 26:756–766. 10.1016/j.euroneuro.2016.01.005 26875114 10.1016/j.euroneuro.2016.01.005

[CR59] Puigseslloses P, Nadal-Gratacós N, Ketsela G, Weiss N, Berzosa X, Estrada-Tejedor R, Islam MN, Holy M, Niello M, Pubill D, Camarasa J, Escubedo E, Sitte HH, López-Arnau R (2024) Structure-activity relationships of serotonergic 5-MeO-DMT derivatives: insights into psychoactive and thermoregulatory properties. Mol Psychiatry. 10.1038/s41380-024-02506-8 38486047 10.1038/s41380-024-02506-8 PMC11412900

[CR60] Rich-Edwards JW, Maney DL (2023) Best practices to promote rigor and reproducibility in the era of sex-inclusive research. Elife 12:e90623 37917121 10.7554/eLife.90623 PMC10622144

[CR61] Rickli A, Moning OD, Hoener MC, Liechti ME (2016) Receptor interaction profiles of novel psychoactive tryptamines compared with classic hallucinogens. Eur Neuropsychopharmacol 26:1327–1337 27216487 10.1016/j.euroneuro.2016.05.001

[CR62] Rössler AS, Bernabé J, Denys P, Alexandre L, Giuliano F (2006) Effect of the 5-HT2A/2 C receptor agonist DOI on female rat sexual behavior. J Sex Med 3:432–441. 10.1111/j.1743-6109.2006.00240.x 16681468 10.1111/j.1743-6109.2006.00240.x

[CR64] Sherwood AM, Halberstadt AL, Klein AK, McCorvy JD, Kaylo KW, Kargbo RB, Meisenheimer P (2020) Synthesis and biological evaluation of tryptamines found in hallucinogenic mushrooms: Norbaeocystin, Baeocystin, Norpsilocin, and aeruginascin. J Nat Prod 83:461–467. 10.1021/acs.jnatprod.9b01061 32077284 10.1021/acs.jnatprod.9b01061

[CR63] Sherwood AM, Burkhartzmeyer EK, Williamson SE, Baumann MH, Glatfelter GC (2024) Psychedelic-like activity of Norpsilocin analogues. ACS Chem Neurosci 15:315–327. 10.1021/acschemneuro.3c00610 38189238 10.1021/acschemneuro.3c00610 PMC10797613

[CR65] Sierra S, Muchhala KH, Jessup DK, Contreras KM, Shah UH, Stevens DL, Jimenez J, Cuno Lavilla XK, de la Fuente Revenga M, Lippold KM, Shen S, Poklis JL, Qiao LY, Dewey WL, Akbarali HI, Damaj MI, González-Maeso J (2022) Sex-specific role for serotonin 5-HT2A receptor in modulation of opioid-induced antinociception and reward in mice. Neuropharmacology 209:108988. 10.1016/j.neuropharm.2022.108988 35183539 10.1016/j.neuropharm.2022.108988 PMC8934299

[CR66] Strassman RJ (1995) Human psychopharmacology of N,N-dimethyltryptamine. Behav Brain Res 73:121–124. 10.1016/0166-4328(96)00081-2 10.1016/0166-4328(96)00081-2 8788488

[CR67] Strickland JC, Johnson MW (2022) Human behavioral Pharmacology of psychedelics. Advances in Pharmacology (San Diego. Calif) 93:105–132. 10.1016/bs.apha.2021.10.003 10.1016/bs.apha.2021.10.003 35341564

[CR69] Sumner BE, Fink G (1995) Estrogen increases the density of 5-hydroxytryptamine2A receptors in cerebral cortex and nucleus accumbens in the female rat. J Steroid Biochem Mol Biol 54:15–20 7632610 10.1016/0960-0760(95)00075-b

[CR70] Sumner BE, Fink G (1998) Testosterone as well as Estrogen increases serotonin2A receptor mRNA and binding site densities in the male rat brain. Mol Brain Res 59:205–214 9729388 10.1016/s0169-328x(98)00148-x

[CR68] Sumner B, Grant K, Rosie R, Hegele-Hartung C, Fritzemeier K-H, Fink G (2007) Raloxifene blocks estradiol induction of the serotonin transporter and 5-hydroxytryptamine2A receptor in female rat brain. Neurosci Lett 417:95–99 17398000 10.1016/j.neulet.2007.02.039

[CR71] Tylš F, Pálenícek T, Kaderábek L, Lipski M, Kubešová A, Horácek J (2016) Sex differences and serotonergic mechanisms in the behavioural effects of Psilocin. Behav Pharmacol 27:309–320 26461483 10.1097/FBP.0000000000000198

[CR72] Vizeli P, Studerus E, Holze F, Schmid Y, Dolder PC, Ley L, Straumann I, Becker AM, Müller F, Arikci D, Liechti ME (2024) Pharmacological and non-pharmacological predictors of the LSD experience in healthy participants. Translational Psychiatry 14:357. 10.1038/s41398-024-03074-9 39231959 10.1038/s41398-024-03074-9 PMC11374807

[CR73] Vohra HZ, Saunders JM, Jaster AM, de la Fuente Revenga M, Jimenez J, Fernández-Teruel A, Wolstenholme JT, Beardsley PM, González-Maeso J (2022) Sex-specific effects of psychedelics on prepulse Inhibition of startle in 129S6/SvEv mice. Psychopharmacology 239:1649–1664. 10.1007/s00213-021-05913-9 34345931 10.1007/s00213-021-05913-9 PMC10103008

[CR74] Vollenweider FX, Vollenweider-Scherpenhuyzen MF, Bäbler A, Vogel H, Hell D (1998) Psilocybin induces schizophrenia-like psychosis in humans via a serotonin-2 agonist action. NeuroReport 9:3897–3902. 10.1097/00001756-199812010-00024 9875725 10.1097/00001756-199812010-00024

[CR75] Warren AL, Lankri D, Cunningham MJ, Serrano IC, Parise LF, Kruegel AC, Duggan P, Zilberg G, Capper MJ, Havel V, Russo SJ, Sames D, Wacker D (2024) Structural Pharmacology and therapeutic potential of 5-methoxytryptamines. Nature. 10.1038/s41586-024-07403-2 38720072 10.1038/s41586-024-07403-2 PMC11152992

