# Supplementary material for: Psychedelic-like effects induced by 2,5-dimethoxy-4-iodoamphetamine, lysergic acid diethylamide, and psilocybin in male and female C57BL/6J mice
Source: Psychopharmacology (Berl). 2025 May 17;242(10):2249–60. doi: 10.1007/s00213-025-06795-x (PMC12449377; doi:10.1007/s00213-025-06795-x)
Supplement: Supplementary file 1 — Supplementary Material 1 [file 213_2025_6795_MOESM1_ESM.pdf]

Supporting Information

Psychedelic-like effects induced by 2,5-dimethoxy-4-iodoamphetamine, lysergic acid diethylamide, and psilocybin in male and female C57BL/6J mice

Shelby A. McGriff^[1], Jacquelin C. Hecker^[2], Alexander D. Maitland^[1], John S. Partilla[1], Michael H. Baumann[1], Grant C. Glatfelter*[1]

[1]Designer Drug Research Unit, National Institute on Drug Abuse, Intramural Research Program, Baltimore, MD 21224, USA

[2]Behavioral Neuroscience Research Branch, National Institute on Drug Abuse Intramural Research Program, Baltimore, MD 21224, United States

^Contributed equally

*Corresponding author:

grant.glatfelter@nih.gov

Acknowledgements

This work was supported by NIDA Intramural Research Program grant number DA-000522-16 (M.H.B.).

No authors report any conflicts of interest related to the present research.

Table S1. Affinity (Ki) of DOI, LSD, and psilocybin in 5-HT_{1A} and 5-HT_{2A} mouse brain binding assays. m5-HT_{1A} and m5-HT_{2A} = mouse 5-HT_{1A} and 5-HT_{2A}. Data are expressed with 95% confidence intervals noted below in parentheses.

Ligand	Mouse Brain Affinity			
	[^{3}H]8-OH-DPAT Binding ♀ m5-HT_{1A}	[^{3}H]8-OH-DPAT Binding ♂ m5-HT_{1A}	[^{3}H]M100907 Binding ♀ m5-HT_{2A}	[^{3}H]M100907 Binding ♂ m5-HT_{2A}
	K_i (nM) 95% CI	K_i (nM) 95% CI	K_i (nM) 95% CI	K_i (nM) 95% CI
DOI	2,651 (1,941 – 3,653)	2,869 (1,735 – 4,909)	11 (5.6 – 23)	13 (8.3– 20)
LSD	0.95 (0.72 – 1.25)	1.02 (0.69– 1.49)	1.9 (1.5 – 2.4)	2.3 (1.9– 2.9)
Psilocin	100 (76.2 – 134)	113 (87.0 – 146)	113 (87.4 – 146)	110 (82.0 – 148)

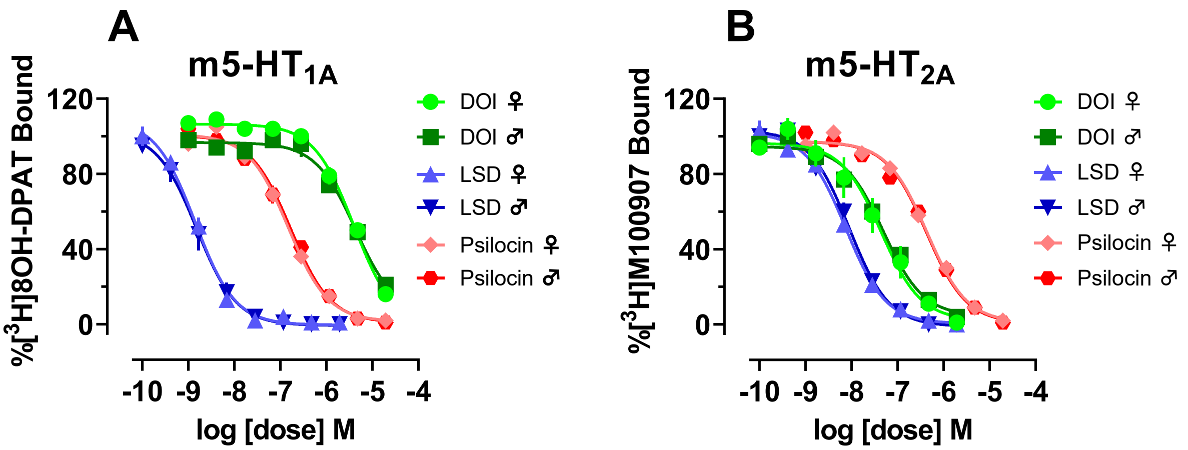

Figure S1. Concentration-response curves for competition of DOI, LSD, and psilocybin for m5-HT_{1A} and m5-HT_{2A} receptor binding.

Table S2. Descriptive statistics and comparisons of mouse behavioral data. Data for each dose represent male or female values in white and pooled data in grey. Overall two-way ANOVA results are listed in **Table 2**. Pooled data for DOI ($W_{6.000, 33.53}$ = 125.1 p < 0.0001, $W_{6.000, 33.39}$ = 3.052 p = 0.0164, $W_{6.000, 35.88}$ = 2.924 p = 0.0200), LSD ($W_{6.000, 33.80}$ = 70.17 p < 0.0001, $W_{6.000, 35.07}$ = 5.809 p = 0.0003, $W_{6.000, 35.75}$ = 6.013 p = 0.0002), and psilocybin ($W_{6.000, 29.79}$ = 27.49 p < 0.0001, $W_{6.000, 30.63}$ = 20.67 p < 0.0001, $W_{6.000, 28.92}$ = 40.98 p < 0.0001) are shown in grey shaded rows and compared via Welch's one-way ANOVA with Dunnett's T3 multiple comparisons tests vs. vehicle controls (0 mg/kg). Bold values and post test values designate significant differences in sex x dose (white rows) or dose vs. vehicle post tests (grey rows).

**Drug *n* per dose**	**Dose**	**HTR (total events)**		**Post Test**	**Body Temp Δ (°C)**		**Post Test**	**Locomotor Activity (cm)**		**Post Test**
	**mg/kg**	**Mean**	**SEM**	***p* value**	**Mean**	**SEM**	***p* value**	**Mean**	**SEM**	***p* value**
DOI *n* = 5 – 7	0 ♂	7.4	1.3	-	-0.04	0.1	-	2669	382.7	-
	0 ♀	7.3	1.6	>0.9999	-0.1	0.2	>0.9999	2512	239.2	>0.9999
	0	7.4	0.9	-	-0.1	0.1	-	2591	217.9	-
	0.03 ♂	8.8	0.3	-	0.2	0.1	-	2753	238.9	-
	0.03 ♀	12.5	1.3	>0.9999	0.0	0.1	0.9952	2149	244.0	0.8575
	0.03	10.7	0.9	0.1000	0.1	0.1	0.6843	2408	186.0	0.9866
	0.1 ♂	22.4	2.2	-	0.3	0.1	-	3045	200.8	-
	0.1 ♀	19.7	1.1	>0.9999	0.2	0.1	>0.9999	2292	293.5	0.7512
	0.1	**20.8**	**1.2**	**<0.0001**	-0.1	0.1	>0.9999	2606	214.6	>0.9999
	0.3 ♂	57.3	7.2	-	0.6	0.2	-	3242	331.5	-
	0.3 ♀	59.0	3.5	>0.9999	0.2	0.1	0.9999	3289	513.0	>0.9999
	0.3	**58.2**	**3.8**	**<0.0001**	0.2	0.1	0.1353	3165	234.1	0.3948
	1 ♂	89.7	6.1	-	-0.1	0.2	-	4033.6	454.7	-
	1 ♀	91.2	5.8	>0.9999	-0.3	0.1	0.8826	3246.7	331.5	0.6942
	1	**90.4**	**4.0**	**<0.0001**	0.4	0.1	0.0724	3640	293.3	0.0513
	3 ♂	106.8	12.4	-	-0.1	0.4	-	3289.3	513.0	-
	3 ♀	108.8	8.4	>0.9999	-0.3	0.3	0.9949	3192.6	539.2	>0.9999
	3	**107.8**	**7.1**	**<0.0001**	-0.2	0.2	0.9948	3241	355.1	0.5495
	10 ♂	**103.3**	**20.3**	**-**	-0.9	0.7	-	2529.1	641.8	-
	10 ♀	**148.7**	**14.2**	**0.0017**	-0.4	0.2	0.6537	2459.7	312.0	>0.9999
	10	**126.0**	**13.7**	**<0.0001**	-0.6	0.3	0.5315	2492	324.8	>0.9999
LSD *n* = 6	0 ♂	6.5	1.5	-	-0.1	0.1	-	2508.0	331.3	-
	0 ♀	9.7	2.2	0.9837	-0.3	0.1	0.9851	2297.1	230.1	0.9944
	0	8.1	1.4	-	-0.2	0.1	-	2403	194.9	-
	0.003♂	6.3	1.3	-	-0.1	0.1	-	2549.1	311.2	-
	0.003♀	6.5	0.9	>0.9999	-0.1	0.2	>0.9999	2499.8	281.8	>0.9999
	0.003	6.4	0.8	0.8589	-0.1	0.1	0.9825	2526	203.4	0.9981
	0.01 ♂	13.5	1.3	-	0.3	0.1	-	2649.3	127.8	-
	0.01 ♀	10.7	1.2	0.9915	-0.1	0.1	0.6367	2581.2	218.0	>0.9999
	0.01	12.1	0.9	0.1403	0.1	0.1	0.1868	2615	120.9	0.9194
	0.03 ♂	29.7	1.8	-	0.0	0.1	-	2756.4	275.3	-
	0.03 ♀	24.2	2.0	0.7203	-0.1	0.1	0.9996	2977.4	152.5	0.9884
	0.03	**27.2**	**1.5**	**<0.0001**	-0.1	0.1	0.7973	2867	154.3	0.3534

	0.1 ♂	43.7	2.0	-	-0.1	0.2	-	2650.0	184.8	-
	0.1 ♀	43.5	7.1	>0.9999	-0.3	0.1	0.9569	2290.0	139.4	0.8787
	0.1	**43.6**	**3.5**	**<0.0001**	-0.2	0.1	0.9996	2456	120.3	0.999
	0.3 ♂	45.0	2.6	-	-0.1	0.3	-	2123.8	180.5	-
	0.3 ♀	55.8	4.6	0.0708	-0.4	0.2	0.5851	2309.5	294.8	0.9975
	0.3	**50.4**	**2.9**	**<0.0001**	-0.2	0.2	0.9992	2217	167.2	0.9743
	1 ♂	**26.2**	**2.6**	**-**	-1.5	0.3	-	1571.6	162.0	-
	1 ♀	**38.0**	**3.1**	**0.0368**	-0.9	0.1	0.1234	2029.2	122.0	0.7257
	1	**32.1**	**2.6**	**<0.0001**	**-1.2**	**0.2**	**0.0014**	1800	118.8	0.0906
Psilocybin<br>*n* = 5 – 6	0 ♂	6.5	1.2	-	-0.3	0.2	-	2430.0	179.3	-
	0 ♀	6.8	1.8	>0.9999	-0.03	0.2	0.9907	2356.1	279.8	>0.9999
	0	6.7	1.1	-	-0.1	0.2	-	2396	152.1	-
	0.03 ♂	7.6	2.5	-	-0.4	0.1	-	2063.8	196.3	-
	0.03 ♀	8.0	0.9	>0.9999	0.2	0.1	0.7834	2480.4	322.6	0.8843
	0.03	7.8	1.2	0.9781	-0.1	0.1	>0.9999	2272	191.1	0.9956
	0.1 ♂	13.0	2.0	-	0.3	0.3	-	2877.2	260.3	-
	0.1 ♀	11.8	1.6	>0.9999	0.1	0.04	0.9997	2686.5	415.1	0.9986
	0.1	**12.4**	**1.2**	**0.0127**	0.2	0.1	0.5729	2782	233.1	0.6695
	0.3 ♂	26.8	2.8	-	0.1	0.1	-	3064.5	297.1	-
	0.3 ♀	27.3	4.2	>0.9999	0.04	0.1	>0.9999	3293.8	114.1	0.9922
	0.3	**27.1**	**2.4**	**<0.0001**	0.1	0.1	0.8728	**3179**	**155.6**	**0.0099**
	1 ♂	39.2	4.6	-	-1.0	0.5	-	3532.9	209.8	-
	1 ♀	30.2	2.0	0.4091	0.0	0.2	0.0935	2900.9	222.6	0.4408
	1	**34.7**	**2.8**	**<0.0001**	-0.4	0.3	0.8733	**3188**	**177.2**	**0.0168**
	3 ♂	**29.4**	**4.3**	**-**	-1.3	0.3	-	2018.9	375.4	-
	3 ♀	**47.2**	**7.4**	**0.0042**	-1.0	0.3	0.9950	2041.8	355.7	>0.9999
	3	**38.3**	**5.0**	**0.0006**	**-1.2**	**0.2**	**0.0034**	2029	245.4	0.7421
	10 ♂	18.7	3.4	-	-4.1	0.7	-	741.5	100.3	-
	10 ♀	21.8	3.0	0.9902	-3.3	0.2	0.3161	962.1	152.5	0.9938
	10	**20.3**	**2.2**	**0.0003**	**-3.7**	**0.4**	**<0.0001**	**851.8**	**93.15**	**<0.0001**

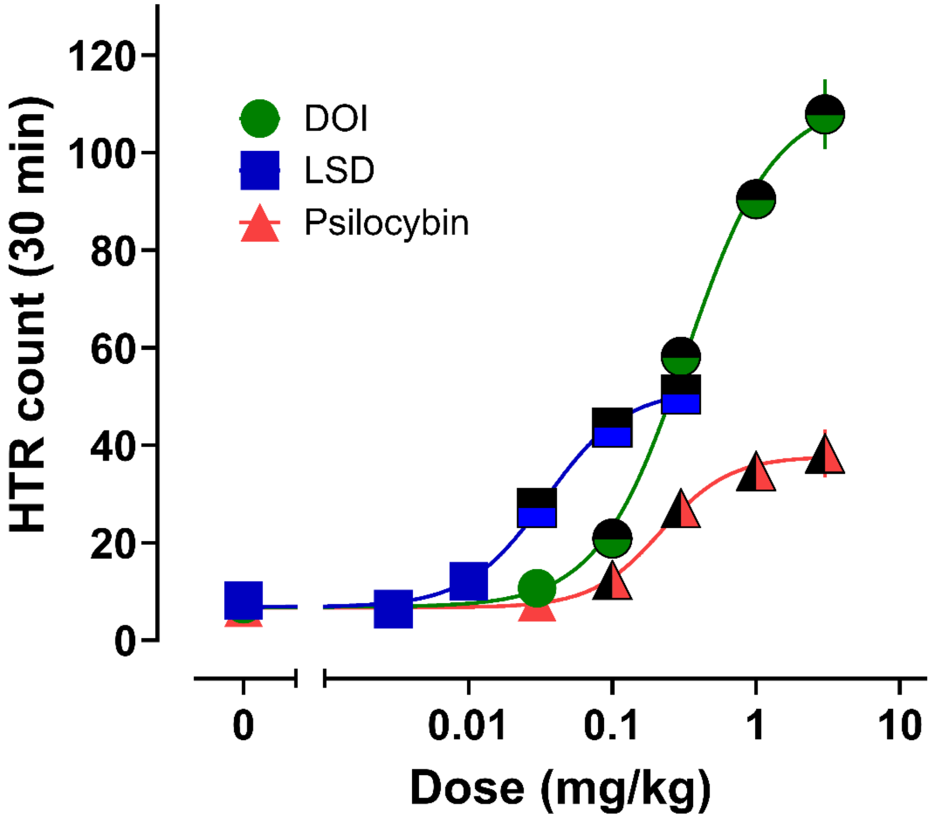

Figure S2. Pooled data rising phase dose-response curves for acute effects of DOI, LSD, and psilocybin on HTR. Descriptive statistics are listed in **Table S2**.

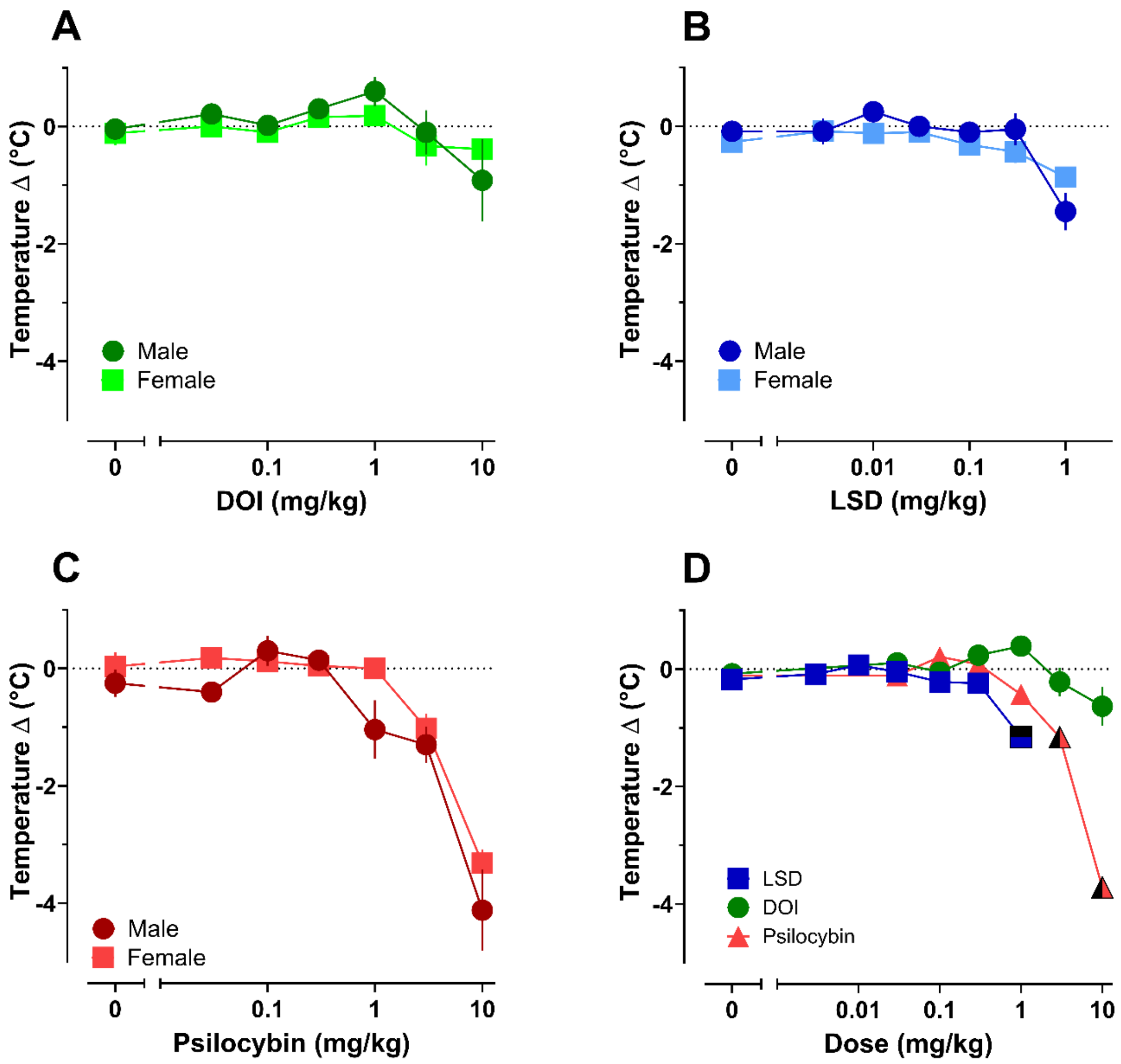

Figure S3. Dose-response curves for acute effects of DOI, LSD, and psilocybin on thermoregulation. Descriptive statistics are listed in **Table S2**. Half-filled symbols represent significant differences vs. 0 mg/kg ($p < 0.05$).

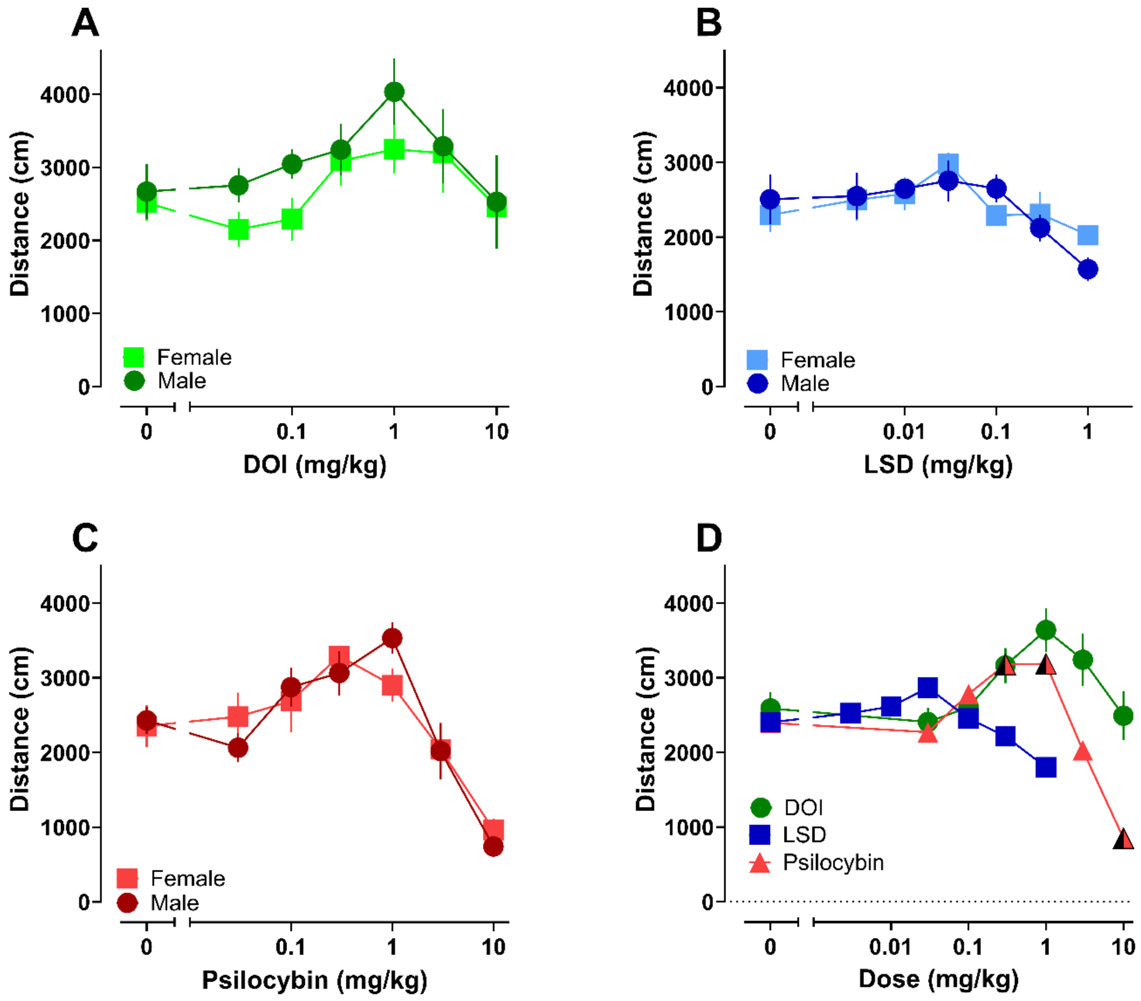

Figure S4. Dose-response curves for acute effects of DOI, LSD, and psilocybin on locomotor activity. Descriptive statistics are listed in **Table S2**. Half-filled symbols represent significant differences vs. 0 mg/kg ($p < 0.05$).

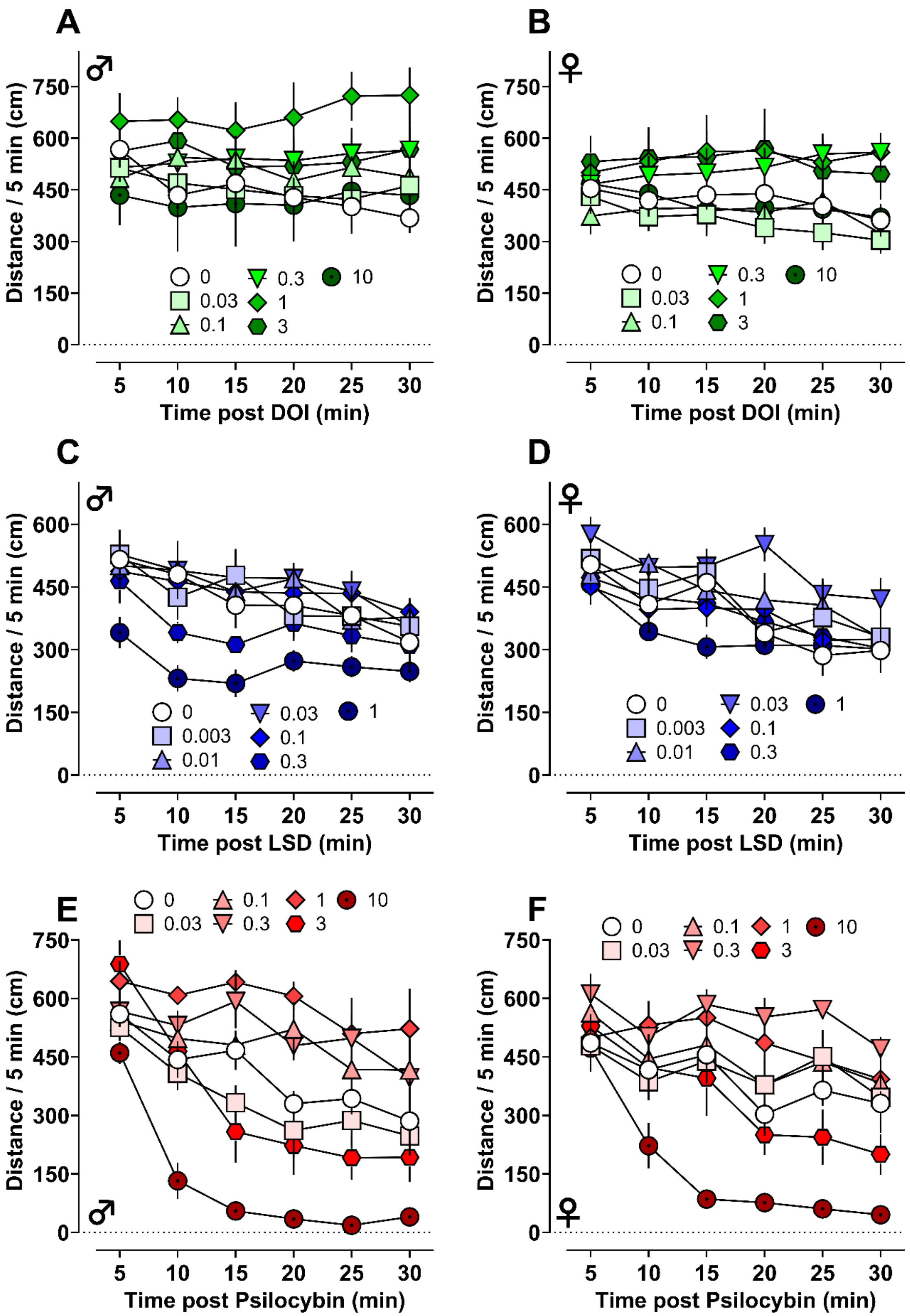

Figure S5. Time-course for acute effects of DOI, LSD, and psilocybin on locomotor activity.

Table S3. Summary of overall male vs. female statistical comparisons for HTR data for DOI, LSD, and psilocybin using a two-way ANOVA.

**Drug**	**Variable**	**Overall Two-way ANOVA (sex x dose)**	***p* value**
DOI	Sex	F (1, 72) = 2.790	0.0992
	Dose	F (6, 72) = 71.48	< 0.0001
	Dose x Sex Interaction	F (6, 72) = 2.083,	0.0657
LSD	Sex	F (1, 71) = 2.593	0.1118
	Dose	F (6, 71) = 72.38	< 0.0001
	Dose x Sex Interaction	F (6, 71) = 2.631	0.0232
Psilocybin	Sex	F (1, 62) = 0.9137	0.3429
	Dose	F (6, 62) = 28.22	< 0.0001
	Dose x Sex Interaction	F (6, 62) = 2.683	0.0222

Table S4. Summary of overall male vs. female statistical comparisons for temperature change and locomotor activity data for DOI, LSD, and psilocybin using a two-way ANOVA.

**Drug**	**Measure**	**Variables**	**Overall Two-way ANOVA (sex x dose)**	***p* value**
DOI	Temp	Sex	$F\ (1, 74) = 0.4770$	0.4919
		Dose	$F\ (6, 74) = 3.474$	0.0044
		Dose x Sex Interaction	$F\ (6, 74) = 0.6871$	0.6606
LSD	Temp	Sex	$F\ (1, 73) = 1.099$	0.2980
		Dose	$F\ (6, 73) = 11.40$	< 0.0001
		Dose x Sex Interaction	$F\ (6, 73) = 1.845$	0.1021
Psilocybin	Temp	Sex	$F\ (1, 64) = 5.817$	0.0188
		Dose	$F\ (6, 64) = 45.36$	< 0.0001
		Dose x Sex Interaction	$F\ (6, 64) = 1.147$	0.3459
DOI	Locomotor	Sex	$F\ (1, 75) = 3.413$	0.0686
		Dose	$F\ (6, 75) = 2.878$	0.0141
		Dose x Sex Interaction	$F\ (6, 75) = 3.597$	0.9020
LSD	Locomotor	Sex	$F\ (1, 74) = 0.04262$	0.8370
		Dose	$F\ (6, 74) = 4.372$	0.0008
		Dose x Sex Interaction	$F\ (6, 74) = 0.7498$	0.6115
Psilocybin	Locomotor	Sex	$F\ (1, 64) = 5.9 \times 10^{-5}$	0.9939
		Dose	$F\ (6, 64) = 22.42$	< 0.0001
		Dose x Sex Interaction	$F\ (6, 64) = 0.9397$	0.4735

AUTHOR INFORMATION

Corresponding Author

Grant C. Glatfelter - Designer Drug Research Unit, National Institute on Drug Abuse Intramural Research Program, Baltimore, MD 21224, United States

grant.glatfelter@nih.gov

Authors

Shelby A. McGriff - Designer Drug Research Unit, National Institute on Drug Abuse Intramural Research Program, Baltimore, MD 21224, United States

Jacquelin C. Hecker – Behavioral Neuroscience Research Branch, National Institute on Drug Abuse Intramural Research Program, Baltimore, MD 21224, United States

Alexander D. Maitland - Designer Drug Research Unit, National Institute on Drug Abuse Intramural Research Program, Baltimore, MD 21224, United States

John S. Partilla - Designer Drug Research Unit, National Institute on Drug Abuse Intramural Research Program, Baltimore, MD 21224, United States

Michael H. Baumann - Designer Drug Research Unit, National Institute on Drug Abuse Intramural Research Program, Baltimore, MD 21224, United States
